# Detection of C-Reactive Protein Using Histag-HRP Functionalized Nanoconjugate with Signal Amplified Immunoassay

**DOI:** 10.3390/nano10061240

**Published:** 2020-06-26

**Authors:** Mohd Farhan Siddiqui, Zeeshan Ahmad Khan, Seungkyung Park

**Affiliations:** School of Mechanical Engineering, Korea University of Technology and Education, Cheonan 31253, Korea; siddiquifarhan87@gmail.com (M.F.S.); acezeeshan@live.com (Z.A.K.)

**Keywords:** Histag-HRP nanoconjugate, immunoassay, C-reactive protein, ELISA, biosensor

## Abstract

Ultrasensitive detection of biomarkers is highly significant for disease prognosis and public health treatment. Despite wide acceptance in routine laboratory tests, the conventional enzyme-linked immunosorbent assay (ELISA) has been of limited use for early biomarker detection due to insufficient sensitivity and multiple long incubation time. Several nanoprobes have been introduced to circumvent the limitation, however, rapid, simple, and chemical-free nanoprobe synthesis and sensitive detection methods, particularly for ELISA, are still lacking. In this study, we have synthesized a gold nanoprobe, conjugated with multiple 6X-histidine (6X-his) peptide and nickel-horseradish peroxidase (Ni^2+^-HRP), for enhancing the colorimetric signal in ELISA. The developed nanoprobe has been tested for the detection of immunologically significant C-reactive protein (CRP) in ELISA format. The performance of designed probe is validated by testing standard and serum samples, and the detection limit of 32.0 pg/mL with R^2^ = 0.98 is confirmed. Furthermore, a comparative analysis of the developed nanoprobe was performed with ELISA developed on conventional guidelines, the proposed immunoassay showed an increase of 12-fold sensitivity for detecting CRP due to the high loading of 6Xhis peptide and binding of multiple Ni^2+^-HRP on a gold nanoparticle. Additionally, the proposed assay provides a simple, fast, and cost-efficient (not requiring multiple antibodies) detection of CRP with easy nanoprobe synthesis. Moreover, the developed Histag-HRP functionalized nanoconjugate immunoassay is flexible and can be applied to other biomarkers efficiently by using disease specific antibody.

## 1. Introduction

C-reactive protein (CRP), an acute-phase protein (23 KDa), is regarded as a significant biomarker for various inflammatory responses. Overexpression of CRP is associated with poor outcomes in various disorders such as cancers, cardiovascular diseases (CVD), diabetes, and neonatal sepsis. CRP is being recently considered as a prognostic biomarker of cardiovascular risk due to its high correlation with the early stage CVD. Therefore, routine monitoring of CRP is crucial, not only for diagnostics but also for proper disease management [1,2,3]. In general, primary prevention for CVD, CRP levels <9 nM, 9–40 nM, and >40 nM are associated with low, moderate, and high risk, respectively. Therefore, to target CVD at an early stage, a highly sensitive method is required that can detect CRP level in pg/mL [4,5,6].

The conventional methods such as nephelometric assays, enzyme-linked immunosorbent assay (ELISA), surface plasmon resonance, electrochemical, chemiluminescence, and fluorescence assay have been introduced for the estimation of CRP [7,8]. In terms of overall cost and simplicity, ELISA has been considered as the most effective and powerful method [9,10,11]. Despite the wide clinical acceptance of ELISA, sensitivity in the range of ng/mL–µg/mL limits its application especially in the early detection of specific biomarkers. Additionally, the use of multiple antibodies and their long incubation time complicates the assay process and limits the sensitivity [12,13,14,15].

“In sandwich ELISA, sensitivity depends on the binding affinity of (1) two primary monoclonal antibodies with different epitopes against the same antigen. One of them is used as capture antibody that is used to coat the polystyrene wells and the other primary antibody is used for detection the antibody, thus, making a sandwich of antibody and antigen. (2) Reporter conjugated-secondary antibody, which is complementary to the detector primary antibody and multiple secondary antibody binds with the primary antibody and thus, amplifies the signal. More antibody, complementary to secondary antibody can also be utilized to further enhance the signal” [16,17]. Conjugating the secondary or tertiary antibody with the reporter molecule is a predominantly used strategy for enhancing sensitivity [18]. In several ELISA protocols, horseradish peroxidase (HRP), a reporter molecule labeled with secondary antibody, is responsible for generating the signals. Improving the number of HRP molecule on the antibody could be the easiest target for enhancing the signal sensitivity. The conventional ELISA signals are generated through 1:1 HRP-secondary antibody binding ratio, which can cause insufficient signal generation leading to poor sensitivity for early biomarker screening [19,20]. Some modifications, such as introduction of streptavidin and biotin-conjugated beads, and metal nanoparticles conjugates have been adopted to improve this binding ratio. Particularly, metal nanoparticles are utilized because of the high surface to volume ratio. Among the various metal nanoparticles such as magnetic, silver, quantum dots, and gold nanoparticles (AuNPs), AuNPs are considered mostly for signal amplification purposes due to their simple synthesis, biocompatibility, and easy electrostatic conjugation with biomolecules [21,22]. AuNPs offered unprecedented opportunity for loading multiple antibodies and reporter molecules on its surface and exhibiting enhanced signals, resulting in achieving the sensitivity of up to 1 ng/mL for CRP detection [18,23]. However, further enhancement of the assay sensitivity in pg/mL range is required for early CRP detection along with maintaining assay cost and complexity for wide acceptance.

In this study, we have designed a novel Histag-HRP functionalized nanoconjugate based immunoassay for CRP detection with enhanced signal sensitivity and simplicity. The strategy is based on the concept of conjugating the high affinity of a peptide (6Xhis) with a detector molecule (Ni-HRP) on the surface of AuNPs. Currently, peptides are considered to be interesting due to low cost, low molecular weight, and easy synthesis for biomedical applications [24]. A smaller size than an antibody, in particular, is the key property that can allow each peptide to bind in closer proximity on the surface of a gold nanoparticle, and thus, increases the loading capacity and sensitivity of the assay. Furthermore, superior affinity and specificity than antibodies are additional advantages that are making them suitable for diagnostics applications [25]. Inspired by these properties of peptides, we have synthesized a peptide nanoprobe and designed an immunoassay for the detection of CRP with amplified signals. In general, 6Xhis peptide can bind with three molecules of Ni-HRP, by conjugating his-Ni^2+^HRP complex on the surface of gold nanoparticle, the overall loading of HRP molecules could be increased, which can enhance the signal sensitivity. To validate the hypothesis, the performance of the developed nanoprobe is experimentally evaluated and compared with conventional ELISA using standard solution as well as human serum, and the overall time of assay and cost of the proposed device are discussed.

## 2. Experimental Procedure

### 2.1. Preparation of the 6XHis-Gold-HRP Nanoprobes

The 6XHis nanoprobe (15 nm) was prepared in water using a simple mixing and centrifugation approach. A 1:1 ratio (*w*/*w*) of antibody and 6Xhis (NH_2_-H-H-H-H-H-H-COOH; 6XHistag^®^ peptide, ab14943, Abcam, Cambridge, UK) was used for the conjugation on the AuNPs. In the first step, 20 µL of anti-CRP-monoclonal antibody (abCRP; 5 mg/mL, Thermo scientific, Waltham, MA, USA) was mixed with 1 mL AuNPs (2.7 nM, 15 nm) incubated for 20 min at room temperature (RT) under vigorous stirring (500 rpm). The unbound abCRP was removed from the reaction mixture by centrifugation (10,000 rpm for 20 min) at 4 °C, followed by re-suspension of the antibody-AuNPs in 1 mL ultrapure water. In the next step, 100 µL 6Xhis (10 µg/mL, Abcam, Cambridge, UK) were added to the antibody-AuNPs suspension. The suspension was then rotated at 30 rpm for 30 min at room temperature. After the stirring, the reaction mixture was centrifuged at (10,000 rpm for 15 min) at 4 °C. Subsequently, the supernatant was collected and incubated in 100 µL of 3% casein (Sigma-Aldrich, St. Louis, MO, USA) for blocking nonspecific binding. The suspension was then stirred for another 15 min at 500 rpm, followed by centrifugation at 10,000 rpm for another 15 min. The precipitated gold nanoprobe was resuspended in water and stored at 4 °C. Afterward, the 100 µL of 1 µg/mL nickel (Ni^2+^) activated horseradish peroxidase (HisProbe™-HRP Conjugate, Thermo scientific, Waltham, MA, USA) probe was incubated with the 6Xhis-AuNp-abCRP suspension for 30 min at 30 rpm under RT. The incubated sample was washed twice by centrifugation for the 10 min interval at 10,000 rpm. Finally, the palette of gold nanoprobe was collected, suspended in ultrapure water, and stored at 4 °C for further use.

After every step of nanoprobe synthesis, spectrophotometric analysis was carried out using a microplate reader. The reference beam sample was water or an appropriate buffer. Spectra were obtained from 500 to 550 nm (spectral resolution 5 nm). The bare colloid gold solution was used as a blank for non-functionalized surface, and test buffer for functionalized nanoprobes surface with different modifications. AuNPs solutions were monitored for red-shift by measuring and determining the increased size with no antibody, after the addition of the antibody, and after the addition of 6Xhis.

### 2.2. Estimation of 6XHis-HRP Conjugated on a Gold Nanoparticle

To determine the number of the HRP-His attached to the AuNPs surface, HRP-TMB reaction-based colorimetric method was applied. A 100 µL of 6Xhis peptide in the concentration range of 0.2–200 nM was dispensed to each well aliquoted in a coating buffer. The plate was incubated overnight at 4 °C and then blocked with 3% casein. Thereafter, 100 µL of Ni-HRP added to each well and incubated for 30 min at room temperature. Wash each well thrice with wash buffer followed by dispensing 100 µL of by 3,3′,5,5′-tetramethylbenzidine substrate (TMB, Thermo scientific) and hydrogen peroxide. Finally, the plate was incubated, and the reaction was stopped by adding 50 µL of 2 M sulphuric acid (Samchun chemicals, Seoul, Korea). The color intensity was measured at 450 nm through a microplate reader and plot the standard graph between absorbance and his concentration. The concentrations of unknown 6Xhis were determined by interpolation from a standard curve. The average number of oligopeptide per AuNPs was calculated by dividing the concentration of oligopeptide by the concentration of AuNPs.

### 2.3. Enzyme-6Xhis Nanoprobe Immunoassay for the Detection of CRP

The 6Xhis nanoprobe assay is as followed, firstly 100 µL of CRP monoclonal antibody (abCRP’, Abnova, Taibei, Taiwan) diluted in carbonate-bicarbonate buffer (0.1 M, pH 9.6, Duksan chemicals, Ansan, Korea) was coated on polystyrene plate (Nunc Maxisorp, Thermo scientific, Waltham, MA, USA). Thereafter the plate was incubated overnight at 4 °C. The plate was then blocked with 3% casein prepared in phosphate buffer saline (PBS) and 0.05% Tween-20 and incubated for 30 min at 37 °C. Thereafter, 100 µL of the standard solution of C-reactive protein (Thermofisher, Waltham, MA, USA) and spiked human serum samples (Sigma-Aldrich, St. Louis, Missouri United States) with varying concentration (0, 10, 25, 50, 100, 250, 500, 1000 and 2000 pg/mL) was dispensed followed by incubation at 37 °C for 30 min and washing with 250 µL PBS (0.05%, Tween-20). Subsequently, the designed HRP-6Xhis-AuNps-abCRP probe was dispensed and incubated for another 1 h in dark at room temperature. Each well was washed with 250 µL PBST (0.05%, Tween-20) and treated with 100 µL TMB substrate. The plates were then incubated for 10 min at RT under dark. After incubation, the 100 µL 2 M H_2_SO_4_ stop solution was added to each well. Finally, absorbance was measured at 450 nm by the microplate reader.

### 2.4. Conventional Enzyme-Linked Immunosorbent Assay (ELISA) for CRP

The sandwich assay was performed following the protocol published elsewhere [26,27,28]. Briefly, the abCRP’ was coated on 96 well plate and incubated for overnight at 4 °C followed by blocking with 1 mg/mL BSA (single purified protein) and incubated for 1 h at 37 °C. A 100 µL [29]. CRP sample (0–50 ng/mL) spiked in human serum was added to each well and incubated at 37 °C for 2 h. After incubation, the plate was washed with 200 µL PBS wash buffer. 100 µL anti-rabbit IgG–HRP (Santa Cruz Biotechnology, Dallas, TX, USA) was added into each well and then incubated for 2 h in dark at room temperature. Thereafter, each well was rinsed thoroughly with 200 µL PBS. Subsequently, 100 µL TMB was dispensed and incubated for another 30 min in dark at RT. The reaction was stopped by adding 100 µL 2 M H_2_SO_4_ stop solution. The absorbance value from each well was measured at 450 nm by the microplate reader.

## 3. Result and Discussions

### 3.1. Development of HRP-Labeled 6Xhis Nanoprobe

**Scheme 1:**
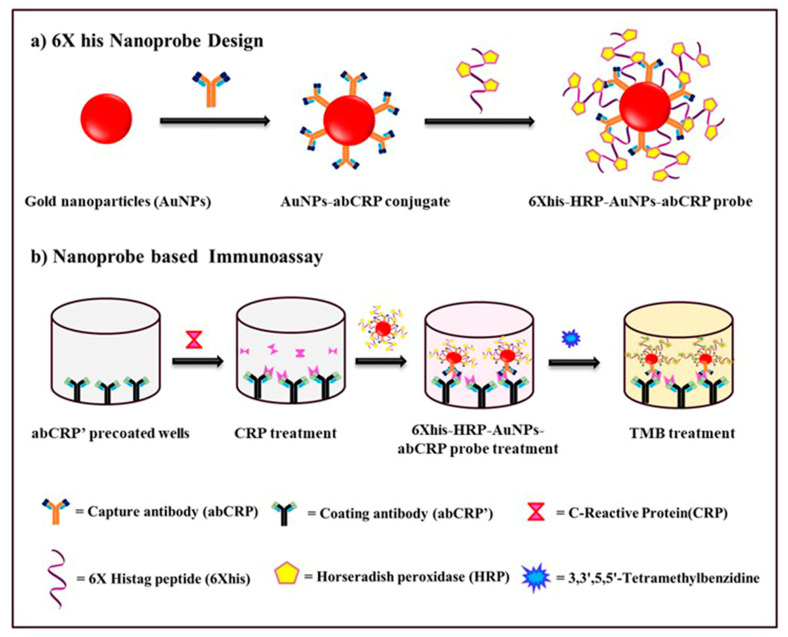
Schematic illustration of procedure for nanoprobe synthesis and colorimetric sensing of CRP detection through HRP-6XHis-AuNPs-anti-CRP probe. (**a**) 6X Nanoprobe Design; (**b**) Nanoprobe based Immunoassay.

For the signal amplification in immunoassay, a 6Xhis peptide-Ni^2+^-HRP nanoprobe with enhanced loading capacity has been developed. 6Xhis peptide contains six residues of amino acid histidine that can specifically bind to Ni^2+^ tagged HRP due to the high affinity of imidazole ring of histidine towards Ni^2+^. Each Ni^2+^ coordinates with two histidine residues of 6Xhis peptide to form a stable complex. In total, three molecules of Ni^2+^-HRP can bind with 6Xhis peptide, and thus increasing the present binding ratio of HRP and antibody from 1:1 to 1:3 [30,31]. This ratio can be amplified further if AuNPs are functionalized with multiple 6Xhis. Following the concept, first, we have designed the 6Xhis-AuNPs probe and then developed the ELISA protocol to validate the performance. The process of designing nanoprobe and developing ELISA is highlighted in Scheme 1. We prepared the nanoprobe by functionalizing AuNPs with both, antibody (abCRP) and peptide (6Xhis-Ni^2+^-HRP) to increase the number of HRP molecules per nanoparticle. Antibody and HRP conjugated peptide were linked on the surface of AuNPs via passive absorption through ionic and hydrophobic interaction. Therefore, minimizing the complex and costly process of chemically linking through covalent bonding [32].

For developing the reproducible immunoassay, the prepared nanoprobe has been characterized by evaluating the protein coating capacity, the protein adsorption, and the density of 6Xhis peptide binding on the surface of AuNPs. AuNPs exhibit localized surface plasmon resonance (LSPR) hence absorbance under the specific wavelength of incident light in a range of 500–600 nm can be utilized. This peak absorbance or optical density (OD) correlates with the concentration and size of nanoparticles formed in the solution. The relationship between, maximum absorbance, extinction coefficient (3.64 × 108 L mol^−1^ cm^−1^), and concentration is used to estimate the size and concentration of nanoparticles [33]. To calculate the diameter of our synthesized AuNPs, we used the Haiss equation,

(1) $$d={\left(\frac{A_{spr}\;\left(5.89\times 10^{-6}\right)}{c_{Au}\;exp\left(C_{1}\right)}\right)}^{\frac{1}{C_{2}}}\;\;$$

where $A_{spr}$ is the absorption (AU) at the peak SPR, $c_{Au}$ is the amount of gold used in the synthesis (moles/L), and $C_{1}$ and $C_{2}$ are constants defined by values −4.75, 0.314, respectively. For determining the concentration of AuNPs,

(2) $$N=\frac{A_{450}\times 10^{14}}{d^{2}\left[-0.295+1.36\;exp\left(-{\left(\frac{d-96.8}{78.2}\right)}^{2}\right)\right]}$$

where N is the number density of AuNPs per mL, $A_{450}$ is the absorption (AU) at 450 nm, and d is the average diameter of the AuNPs.

**Figure 1:**
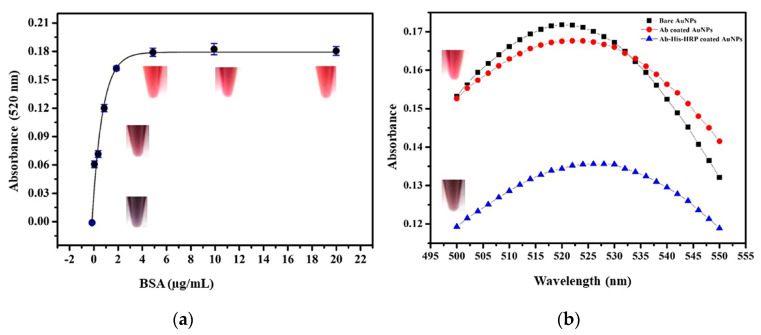
Plot depicting (**a**) the titration of gold nanoparticle with BSA for the binding saturation study, each data point (black dots) represent the error bar, obtained from three sets of experiments. (**b**) UV-Visible characterization of a developed peptide nanoprobe.

The concentration of bare AuNPs and size were estimated to be 2.72 nM and 20 nm (Supplementary Materials Figure S1). Thereafter, titration study was performed using BSA to confer the maximum amount of protein that can be adsorbed on the surface of 100 µL of 20 nm AuNPs, which was inferred from the minimum amount of protein required to stabilize the AuNPs [34]. Figure 1a represented the color change from grey to pink as the BSA concentration increased causing redshift in the absorbance spectra due to subsequent conjugation of BSA on the AuNPs surface. No significant aggregation and color change were observed above 2 µg/mL of BSA. After the estimation of the nanoparticle size, concentration, and maximum protein binding capacity, the antibody, 6Xhis, and Ni^2+^-HRP were sequentially conjugated on the AuNPs. The conjugation was confirmed through quantifying the spectrophotometric changes of AuNPs. As the protein or peptide was conjugated on the AuNPs surface, local refractive index, and absorbance of AuNPs changes. This change was characterized by the redshift in the wavelength of peak absorbance in the visible range [35]. Thus, the developed nanoprobe was simultaneously evaluated by UV-vis-spectrophotometry (400–700 nm) for the subsequent conjugation of CRP antibody and 6Xhis peptide-Ni^2+^-HRP complex. The average diameter of AuNPs before conjugation was estimated to be 20 nm (Supplementary Materials Figure S1) while after conjugation of the abCRP antibody and 6Xhis-Ni-HRP, the size gradually shifted to 24 nm and 28 nm as calculated from the peak obtained at 524 nm and 527 nm, respectively (Equations (1) and (2)). As expected, the spectral shift confirmed the coating of abCRP/6Xhis-Ni^2+^HRP on the surface of AuNPs (Figure 1b).

**Figure 2:**
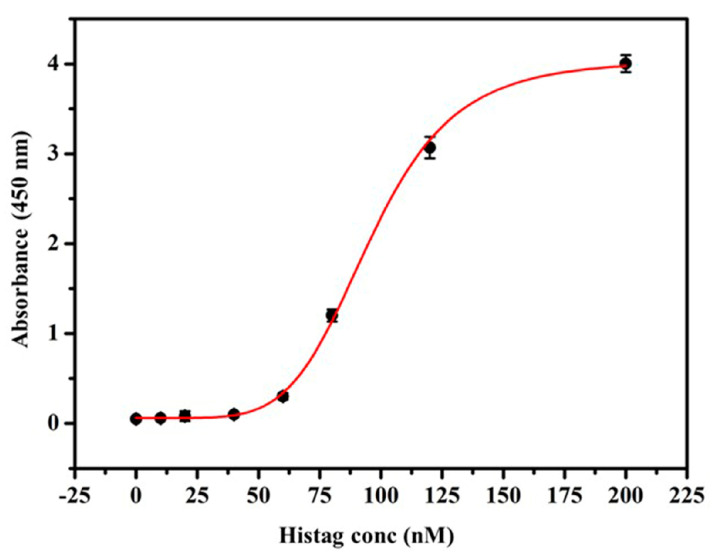
Graphical representation of peptide (6XHis) density estimation conjugated on the surface of gold nanoaprticle. Each data point (black dots) represent the error bar, obtained from three sets of experiments.

After the successful conjugation, the total number of HRP attached to the AuNPs was determined because the HRP is directly responsible for generating the colorimetric signals through TMB oxidation. In conventional ELISA, a 1:1 antibody to HRP ratio is achieved by using a primary or secondary antibody conjugated HRP. This ratio can be increased to 1:3 through streptavidin-biotin chemistry [20,36]. Streptavidin, a 60 kDa tetrameric protein, has a high binding affinity to biotin and four biotin binding sites [16]. Generally, biotinylated antibody and streptavidin-conjugated HRP are used in ELISA to enhance the detection in ELISA [16]. However, the option of further signal enhancement is with streptavidin-biotin chemistry is limited. Thus, instead of using biotinylated antibody and streptavidin-HRP binding chemistry, we have exploited 6Xhis peptide and AuNPs binding chemistry. Multiple 6Xhis can be conjugated on the surface of AuNPs, moreover, one molecule of 6Xhis can bind with three molecules of Ni^2+^-HRP. Then, multiple 6Xhis-Ni-HRP can be conjugated on the surface of AuNPs to further increase the binding ratio to several times. Thus, to understand the strength of signal amplification, the determination of the total number of 6Xhis per nanoparticle is crucial. For this, a standard curve is derived from reactions between different concentrations of 6Xhis-Ni^2+^-HRP and TMB, as shown in Figure 2. From the standard curve, molar extinction coefficient, and concentration of AuNPs, we have calculated the mean density of 6Xhis on each nanoparticle. The average number of 6Xhis Ni^2+^-HRP per nanoparticle was determined to be 30, and hence the overall binding ratio was increased to 1:30. Even though, average number of 6Xhis Ni^2+^-HRP per nanoparticle was determined to be 30 with the aforementioned method, a detailed analysis is required to confirm this number.

### 3.2. Development of Immunoassay

**Figure 3:**
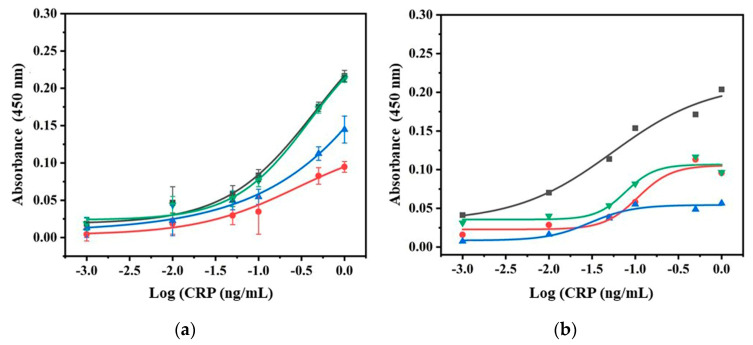
Plot represented the selection of parameters for performing the nanoprobe assay. (**a**) Optimization of capture antibody concentration at three different concentration (2 (red), 3 (blue), 4 (green), and 6 (black) µg/mL), each concentration point is repeated thrice, and (**b**) optimization of probe incubation time at 15 (red), 30 (black), 60 (blue), and 90 (green) min, and 4 µg/mL and 30 min were the finalized capture antibody and incubation time, respectively.

To obtain a better performance, optimization of experimental conditions is crucial. The disproportionate concentration of sample and coating antibody and improper incubation time may result in poor specificity and selectivity, wastage of antibodies, and high background caused by nonspecific binding [34,37]. Therefore, in the present experiment, the concentration of coated abCRP’ and probe reaction time have been studied. Figure 3a showing the detection of CRP at the various concentrations (0–1 ng/mL) with four different concentrations of coated antibody (2, 3, 4, and 6 µg/mL). Detection signal has been observed increasing with the concentration of coated antibody of up to 4 µg/L but saturates above it. Thus, the assay protocol was finalized at 4 µg/L for minimizing the concentration of the antibody. Likewise, choosing the optimum probe incubation time is another important factor as it can negatively influence the sensitivity of the immunoassay. Low incubation time may lead to inefficient binding with the antigen, while an over incubation can increase the background noise signal by hydrophobic binding of immunoglobulin with the bottom and walls of the microwells [38]. The nanoprobe having AuNPs, antibody, and peptide in the ratio of 1:10:50 are used for the selection of incubation time. The probe is incubated at four different intervals of 15, 30, 60, and 90 min with 4 µg/mL coated antibody and 0–1 ng/mL of CRP. Figure 3b shows the effect of incubation time. At lower CRP concentrations, significant differences in responses were observed from 15 to 30 min, which is due to effective binding with antigen over time. When the incubation time was further increased from 60 and 90 min, lower signals were observed probably due to hydrophilic interaction between antibody and microwell, leading to high color intensity in the blank wells. Owing to the high color intensity in the blank well, the difference between the blank and experimental well values decreased. The incubation at 30 min yielded approximately two times higher signal with a clear resolution at a lower concentration. Therefore, 30 min was concluded as the optimum incubation time for nanoprobe based immunoassay for CRP detection.

### 3.3. Analytical Performance of Nanoprobe-ELISA for CRP Detection

**Figure 4:**
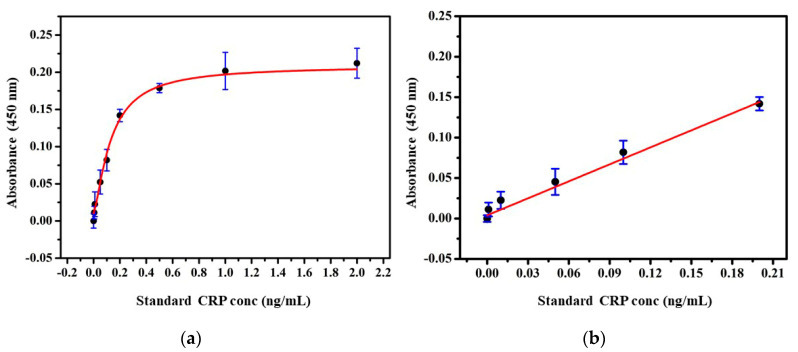
(**a**) Standard graph for detection of CRP based on developed 6XHis nanoprobe. Error bar indicates the standard deviation of four repeated experiments (black dots) and (**b**) along with the fitted standard curve.

Under the optimized condition of nanoprobe and immunoassay, the performance of the developed protocol was examined with standard CRP samples. Varying concentration of CRP from 0 to 2000 pg/mL was employed to produce a calibration curve. In Figure 4a, the absorbance increased as the concentration of CRP increased from 0.01 to 0.5 ng/mL and reached saturation at 1 ng/mL. The linear range of detection between absorbance and CRP concentration was found to be 0−500 pg/mL. The best linear fit for the calibration curve was calculated through the equation Y=0.89232X+0.0039 with $R^{2}=0.985$. In Figure 4b, the limit of detection (LOD) calculated from the curves was recorded to be 0.017 ng/mL. The LOD was estimated as three times the standard deviation (SD) to the slope of the calibration plot (LOD = 3 SD/slope). Compared to the common immunoturbidometric, latex agglutination, or point of care methods, which offered sensitivity in the range of µg/mL, the presented LOD is less than ng/mL, thus, meeting the sensitivity criteria of predictive studies [17].

**Figure 5:**
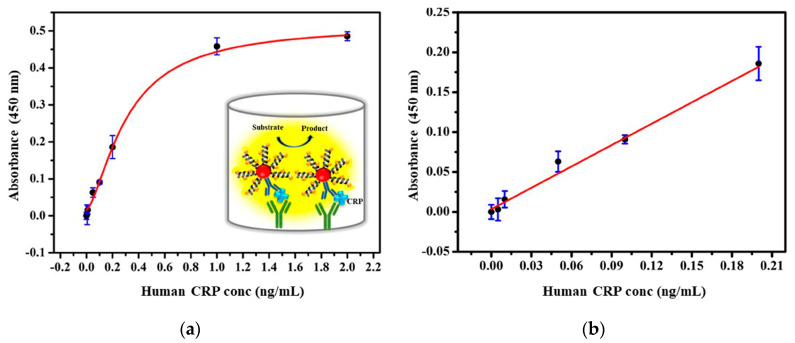
Plot represented the performance of assay (**a**) validation of developed nanoprobe for CRP detection in human serum samples. Error bar indicates the standard deviation of three repeated experiments (black dots) and (**b**) along with the fitted curve.

**Figure 6:**
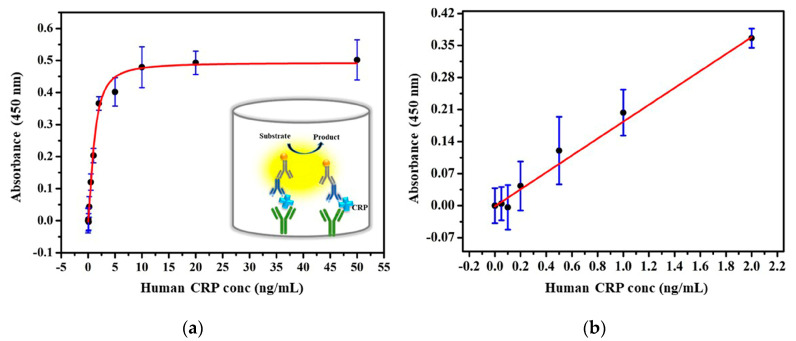
Plot represented (**a**) the performance of classical ELISA. Error bar indicates the standard deviation of three repeated experiments (black dots) and (**b**) along with the fitted curve.

The performance of the developed immunoassay is further evaluated with human serum samples for selective detection in a complex matrix. CRP spiked human serum samples in different concentrations at 0–2 ng/mL were tested with 6Xhis peptide nanoprobe and the results were compared with the traditional ELISA protocol using HRP conjugated secondary antibody as a reference method, as shown in Figure 5 and Figure 6. The developed assay showed the LOD of 0.032 ng/mL, obtained from the best linear fit for the calibration curve *Y* = 0.6999*X* + 0.0039 with R^2^ = 0.984 (*n* = 3; Figure 5). In comparison to the reference ELISA assay, which has shown the LOD of 0.402 ng/mL (Figure 6), the developed assay demonstrated an approximately 12-fold enhancement in the detection limit without the necessity of a secondary antibody. The linear regression of both methods showed a good correlation with R^2^ greater than 0.98. The amplification in the signal sensitivity was directly related to the increased concentration of the HRP substrate on the surface of the nanoparticle. All the OD value results were presented as absolute absorbance obtained after subtracting the OD values from blank wells (blank corrected) [38]. Along with improved sensitivity and simplicity, the present assay provides a reduced incubation time of around 2 h Table 1. Classical ELISA, in general, takes about 4–5 h due to separate primary and secondary antibody incubation steps Table 1 [39].

## 4. Conclusions

In this work, we demonstrated an eco-friendly synthesis of simple, rapid, and sensitive Histag-HRP functionalized nanoconjugate based immunoassay. The designed probe introduced a signal amplification strategy based on the high loading of HRP via 6Xhis-Ni chemistry on the surface of AuNPs. The developed methodology is advantageous in several ways: (1) it eliminates the need of additional detector antibodies, which significantly reduces the cost and processing time, (2) compared to antibodies, the size of the 6Xhis peptide is shorter, which accommodates easily on the surface of gold nanoparticle, thus, minimizing the interference and hindrance, and (3) the conjugation strategy of a peptide nanoprobe is eco-friendly and comparatively rapid than other AuNPs functionalization strategy [40,41]. In summary, we successfully validated our developed nanoprobe and achieved significant signal amplification. The present 6Xhis peptide-based assay performance was efficient in terms of sensitivity, offered a good LOD of 17 pg/mL with standard samples, and 32 pg/mL with human serum samples with a wide linear range of detection (0–0.5 ng/mL). Additionally, we achieved detection within 2 h, which is a significant improvement in performance time as compared to traditional ELISA taking about 5 h to complete the process. Therefore, the proposed Histag-HRP functionalized nanoconjugate based immunoassay provides a promising potential in biomarker estimation for disease diagnosis at an early stage. As future work, detailed analysis of AuNPs conjugated with proteins with Zetasizer and Fourier-transform infrared spectroscopy is required. Moreover, comprehensive validation, and optimization with a wide variety of clinical samples will essentially be required.

## Figures and Tables

**Table 1:** Representing the comparative analysis of developed and conventional ELISA method.

Factors	Developed	Conventional
R^2^	0.984	0.982
Slope	0.669	0.184
Intercept	0.0039	−0.001
LOD (ng/mL)	0.032	0.402
Assay Time	<2 h	>4 h

## Supplementary Materials

The following are available online at https://www.mdpi.com/2079-4991/10/6/1240/s1, Figure S1: SEM characterisation of the synthesized citrate capped gold nanoparticles, the average estimated size is ~20nm.
