# Supplementary material for: Detection of C-Reactive Protein Using Histag-HRP Functionalized Nanoconjugate with Signal Amplified Immunoassay
Source: Nanomaterials (Basel). 2020 Jun 26;10(6):1240. doi: 10.3390/nano10061240 (PMC7353076; doi:10.3390/nano10061240)
Supplement: Supplementary file 1 [file nanomaterials-10-01240-s001.pdf]

Supplementary file:

P-ELISA for Detection of CRP with Amplified Signals Using Gold Nanoprobe

Mohd Farhan Siddiqui†, Zeeshan Ahmad khan† and Seungkyung Park*

School of Mechanical Engineering, Korea University of Technology and Education, Cheonan, 31253, Republic of Korea

* E-mail addresses: spark@koreatech.ac.kr (S. Park)

† These authors contributed equally to this work

Preparation and characterization of gold nanoparticles

Citrate-stabilized gold nanoparticles (AuNPs) were synthesized using trisodium citrate reduction of $HAuCl_4$ through the Turkvich method [30]. Briefly, before use, all glassware used were cleaned with freshly prepared aqua regia (1: 3 HNO_3–HCl), followed by rinsing thoroughly with ultrapure water and dried completely. 500 ul of 38.8 mM trisodium citrate (Sigma-aldrich) was rapidly poured to a 10ml boiling solution of 1 mM $HAuCl_4$ (Sigma aldrich), and the solution was boiled continually with vigorous stirring. The solution was boiled initially for 10 mins and stirred for an additional 15 min to yield a wine-red solution. The solution was cooled at room temperature and filtered by a 0.45 µm membrane. The filtrate was stored in the dark at 4 °C for further use. The synthesize nanoparticle was analyzed through Scanning Electron Microscope (JSM-7500, JEOL Ltd) and images were captured for size estimation **(Supplementary Figure S1).**

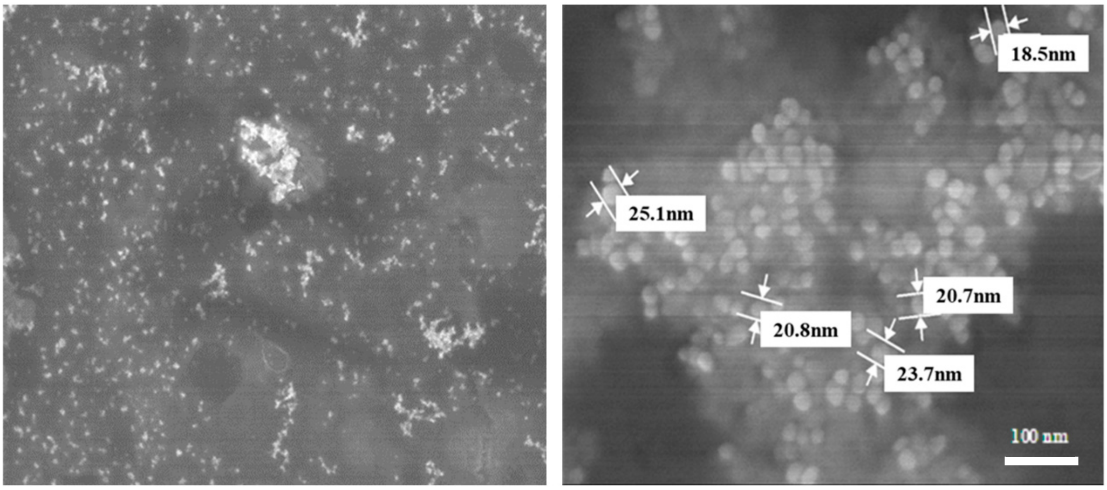

Figure S1: SEM characterisation of the synthesized citrate capped gold nanoparticles, the average estimated size is ~20nm

Titration of gold nanoparticles

96 well microplates were used for titration study of gold nanoparticles. Briefly explained, the 100 µl of synthesized gold nanoparticles was dispensed into each well. In each well, 10 µl of Bovine serum albumin (BSA, Sigma Aldrich) in the range 2-200 µg/ml was added. The plate was then incubated on 37 ^{0}C on orbital shaker for 10 min. Subsequently, 10 µl of 10% aqueous Sodium chloride (NaCl, Thermo scientific) solution was mixed to each well. The aggregation was observed through color change from grey to red, followed by estimating the colorimetric absorbance through microplate reader (Spectra max, Molecular Devices) at wavelength of 520 nm.
